# The National Ambulance Surveillance System: A novel method for monitoring acute alcohol, illicit and pharmaceutical drug related-harms using coded Australian ambulance clinical records

**DOI:** 10.1371/journal.pone.0228316

**Published:** 2020-01-31

**Authors:** Dan I. Lubman, Sharon Matthews, Cherie Heilbronn, Jessica J. Killian, Rowan P. Ogeil, Belinda Lloyd, Katrina Witt, Rose Crossin, Karen Smith, Emma Bosley, Rosemary Carney, Alex Wilson, Matthew Eastham, Toby Keene, Carol Shipp, Debbie Scott

**Affiliations:** 1 Turning Point, Eastern Health, Richmond, Victoria, Australia; 2 Eastern Health Clinical School, Monash University, Box Hill, Victoria, Australia; 3 Monash Addiction Research Centre, Monash University, Frankston, Victoria, Australia; 4 Ambulance Victoria, Doncaster, Victoria, Australia; 5 Department of Community Emergency Health and Paramedic Practice, Monash University, Frankston, Victoria, Australia; 6 Department of Epidemiology and Preventative Medicine, Monash University, Melbourne, Victoria, Australia; 7 Queensland Ambulance Service, Brisbane, Queensland, Australia; 8 New South Wales Ambulance, Rozelle, New South Wales, Australia; 9 Ambulance Tasmania, Hobart, Tasmania, Australia; 10 St John Ambulance Australia (NT) Incorporated, Casuarina, Northern Territory, Australia; 11 Australian Capital Territory Ambulance Service, Fairbairn, Australian Capital Territory, Australia; University of Toronto, CANADA

## Abstract

Although harmful consumption of alcohol and other drugs (both illicit and pharmaceutical) significantly contribute to global burden of disease, not all harms are captured within existing morbidity data sources. Indeed, harms occurring in the community may be missed or under-reported. This paper describes the National Ambulance Surveillance System, a unique Australian system for monitoring and mapping acute harms related to alcohol and other drug consumption. Data are sourced from paramedic electronic patient care records provided by ambulance services from across Australia. Coding occurs in a purpose-built system, by a team of specialised research assistants. Alcohol, and specific illicit and pharmaceutical drugs, rather than broad drug classes, are manually coded and the dataset is reviewed and cleaned prior to analysis. The National Ambulance Surveillance System is an ongoing, dynamic surveillance system of alcohol and other drug-related harms across Australia. The data includes more than 140 output variables per attendance, including individual substances, demographics, temporal, geospatial, and clinical data (e.g., Glasgow Coma Scale score, naloxone provision and response, outcome of attendance). The National Ambulance Surveillance System is an internationally unique population-level surveillance system of acute harms arising from alcohol and other drug consumption. Dissemination of National Ambulance Surveillance System data has been used to inform and evaluate policy approaches and potential points of intervention, as well as guide workforce development needs and clinical practice at the local and national level. This methodology could be replicated in other countries.

## Introduction

Excessive consumption of alcohol and illicit drugs, and extra-medical use of pharmaceutical medications, are major avoidable risk factors for disease, illness, injury and death [1,2]. Globally, 38.3% of the population drink alcohol [3], though this is higher in Australia where, in 2016, 77.5% of adults reported that they consumed alcohol, and 25.5% reported risky drinking [4]. The World Health Organization estimated that approximately five per cent of the global population consumed drugs (substances under the control of international drug control conventions; including both illicit substances and extra-medical pharmaceutical use) at least once in 2015 [5]. Like alcohol, other drug consumption in Australia exceeds global averages, with 12.6% of Australians reporting past-year consumption of an illicit drug in 2016, and 4.8% reported extra-medical use of pharmaceuticals [4]. In the majority of both acute and chronic disease/illness categories, relationships between disease and the volume and/or pattern of alcohol and other drug (AOD) consumption exist, with increasing quantity of alcohol or riskier drug consumption patterns related to higher risk of subsequent disease or death [6].

AOD consumption has been identified as a causal or component risk factor in more than 200 disease and illness categories [6], and resulting harms can be acute or chronic [7]. For example, acute harms include injuries sustained while intoxicated, or unintentional overdose of both illicit and pharmaceutical drugs [8]. In terms of chronic harms, alcohol increases the risk of liver disease, mental disorders, heart disease, some cancers [9], and is the third leading risk factor for premature deaths and disabilities [9]. Chronic stimulant use is associated with increased risk of mental disorders, and opioid consumption is associated with an increased risk of mental disorders and blood borne viruses (associated with injecting drug use) [8]. To understand the impact of AOD consumption in the population, it is important that related harms are measured.

One means of understanding AOD-related harms is burden of disease frameworks, which provide a method for describing and quantifying the health burden of diseases and injuries, and the risk factors (such as AOD) that contribute to them [2,8]. However, not all harms associated with AOD consumption are routinely captured within burden of disease frameworks [10]; for example, many emergency department presentations that involve alcohol may be missed as these are typically classified by the specific presenting disease or injury. Only overdose or poisoning are likely to be captured by primary coding [11], leading to likely underestimation of AOD involvement in these presentations. Additionally, current burden of disease methodologies typically include hospital admission and coronial data, however the impact of AOD consumption on emergency health care responses (such as incidents attended by ambulance) are not currently captured, despite contributing to the overall burden [10].

A second means for quantifying the magnitude of harms is to use survey data. In Australia, instruments for measuring AOD-related harms at a population level include the National Drug Strategy Household Survey (NDSHS) [4] conducted every three years, and targeted surveys (e.g., the Australian Secondary School Drug Survey [12] aimed at students aged 12–18). However, population level surveys have limitations. Although recruitment aims for representative populations, survey frames exclude those outside of the school system or without stable housing. This excludes vulnerable and disadvantaged sub-groups, who may experience greater harms relative to consumption [13]. Additionally, surveys are reliant on self-reported consumption patterns, which are typically described over large intervals (e.g., the past 12 months), and may be prone to recall bias and reporting based on social desirability [14,15]. Furthermore, extremes of consumption within particular population sub-groups, or infrequent high consumption, may be masked by enquiring about average use over a period of time, without taking into account patterns of consumption [16]. Lastly, while instruments such as NDSHS enquire about extra-medical use of pharmaceuticals, medications are broadly categorised into drug classes, and specific medications are not captured [4].

A third means for measuring harms is to analyse administrative data sources, such as emergency department presentation or hospital admission data [17,18]. However, these data, classified using ICD-10, may not be sufficiently detailed to identify trends relative to specific substances [19]. Coronial or mortality data provide an additional source of information on harms [20], but these data only reflect mortality outcomes, and there is a significant reporting lag of two to three years [21]. Though these types of administrative data overcome issues of self-report, not all AOD-related harms will be treated in a hospital setting. For example, opioid poisoning treated with naloxone administration may occur in the community and those affected may not present to hospital, and therefore will not be captured in emergency department or hospital data [22].

Ambulance services are often the first (and frequently primary) contact with health services in the event of an acute AOD-related harm. However, despite ambulance services being available in many countries, their data is not routinely used to capture the breadth of acute AOD-related harms across the community. Ambulance patient care notes offer an additional source of information on acute AOD-related harms that may be missed in other datasets, especially as many of those attended by an ambulance are not transported to hospital. These clinical notes provide an important and rich data source which detail the nature and background to the attendance (including information about what was observed ‘on scene’ such as bystander accounts and evidence of drug paraphernalia), as well as the clinical outcome. In this way, coding paramedic clinical notes addresses an information gap and provides a robust source of information on AOD-related harms. This paper describes the development of the National Ambulance Surveillance System (NASS), an internationally unique system that captures acute harms related to AOD consumption by coding ambulance clinical records, allowing for examination of temporal and spatial trends related to individual substances.

## Materials and methods

### Data coverage and governance

“The Ambo Project”, established in 1998 with funding from the Victorian Department of Health and Human Services, began identifying and classifying AOD (both illicit and pharmaceutical)-related ambulance attendances in metropolitan Melbourne. This initial phase of this work, which primarily focussed on non-fatal heroin overdoses attended by ambulance in Metropolitan Melbourne only [23], served as the basis for NASS. The system underwent a two-phase expansion; in 2011 to incorporate regional Victoria and in 2012 to achieve national coverage, with inclusion of four Australian states (New South Wales, Queensland, Tasmania and Victoria) and two territories (Australian Capital Territory, Northern Territory). NASS captures 82.5% of Australia’s population; with plans to include the two remaining jurisdictions (Western Australia joined the system in 2018 and comparable data provision is anticipated in 2020, and negotiations with South Australia will recommence when an electronical clinical information system becomes available). Coded and categorised data are available for each jurisdiction from the date of jurisdictional project commencement, with complete annual data available for Victoria and data snapshots of one month per quarter (March, June, September and December) available for all other jurisdictions (Table 1). NASS is centrally administered and managed by Turning Point (a national addiction treatment, research and education centre), with data provision governed by agreements with each ambulance service. Rather than focusing on a few sub-types of AOD attendances (e.g., alcohol intoxication or heroin-related), NASS includes both illicit and pharmaceutical drug-related attendances and the inclusion of more than 140 variables, including patient demographics.

**Table 1:** Summary of coded data availability by jurisdiction.

Jurisdiction	Population as at 30 June 2017	Data collection start date	Number of attendances 2016–17 financial year *	Number of AOD-related attendances 2016 calendar year (4 snapshot months) **
Australian Capital Territory	411,667	March 2013	42,098	1,130
New South Wales	7,861,674	January 2013	774,137	13,806***
Northern Territory	247,491	January 2015	33,760	2,883
Queensland	4,929,152	January 2013	767,296	20,780
Tasmania	522,152	March 2013	68,792	1,400
Victoria	6,321,648	November 1998****	497,814	16,541

NB. All dates and numbers are correct as at 30 June 2018.

* Numbers include emergency and urgent incidents, in the Australian financial year (1 July to 30 June)

** Snapshot months: March, June, September, December in 2016 calendar year. Calendar year was used instead of financial year as 2017 data for Queensland is unavailable.

*** This includes cases completed on VACIS^®^, which is over 90% of all attendances

**** Regional Victoria data available from May 2011. Three months of missing data from October to December 2014 inclusive, due to paramedic industrial action

The overall project is approved through the Eastern Health Human Research Ethics Committee (HREC), with additional HREC approval for jurisdictional data provision, and requirements for informed consent were waived by these HRECs. Strict protocols are in place for data de-identification, confidentiality, storage, access and reporting. Patient identifiers are provided by some ambulance jurisdictions for the purposes of data linkage. On data receipt, these identifiers are stripped from the dataset and a unique statistical linkage key (SLK) created. Identifiers are held in a password protected, secure, separate database that is accessible only to database managers. All data is de-identified prior to coding. Of note, NASS spans additional coding modules including ambulance attendances related to mental health, self-harm, and violence.

### Process overview

**Fig 1:**
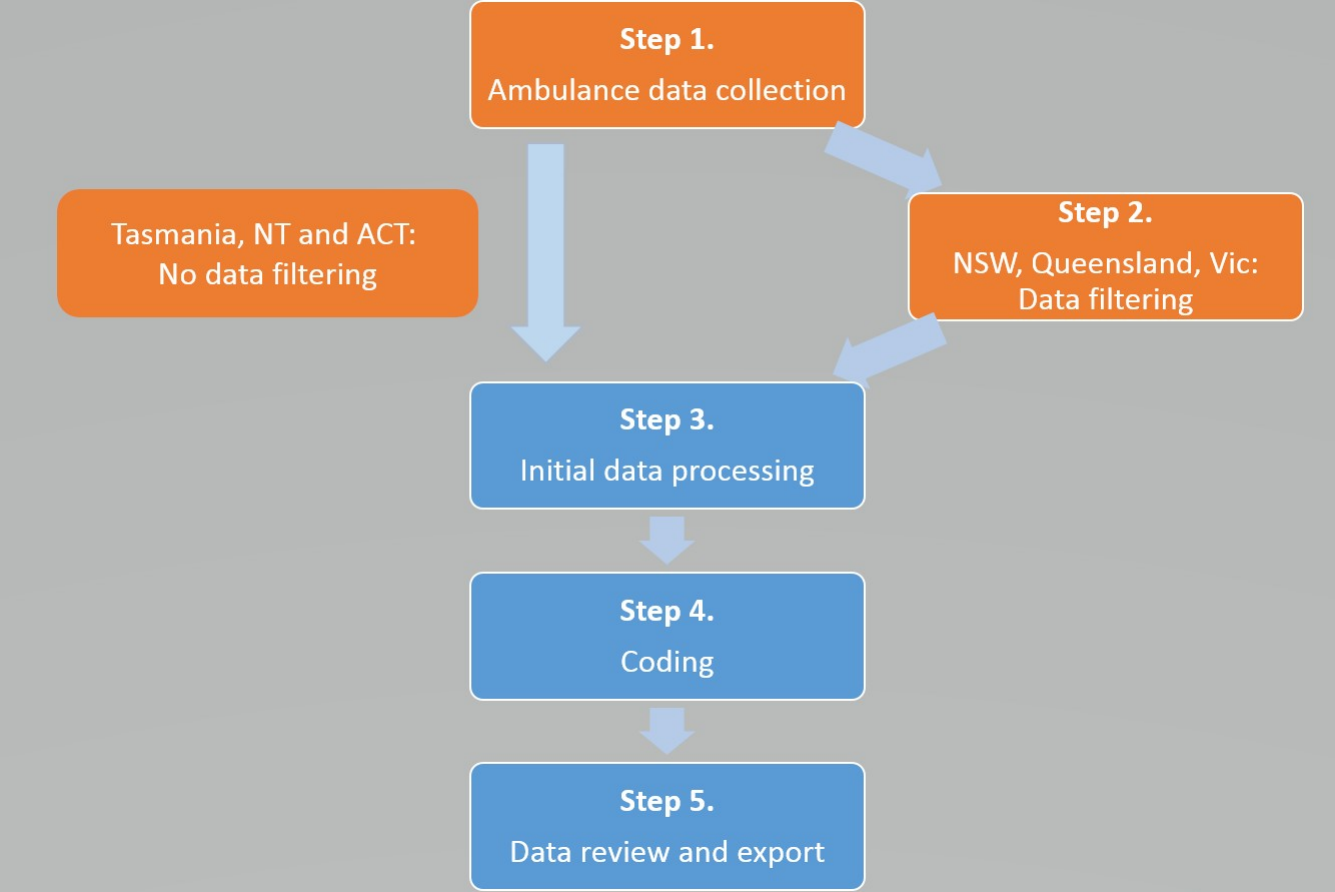
National Ambulance Surveillance System data collection and coding process. Processes in orange occur at the jurisdictional ambulance services, with processes in blue occurring at Turning Point.

Fig 1 presents the five steps in NASS data collection and coding.

#### Step 1 –Ambulance data collection

VACIS^®^ is the clinical record management system used by NASS contributing ambulance services with the exception of the Northern Territory’s use of Siren^®^, and a new system implemented in Queensland in 2017 (2016 Queensland data is reported in this manuscript, which is prior to their system migration). Although system specifics vary slightly the fundamentals remain the same. Paramedics create an electronic patient care record (*e*PCR) for each attendance, which includes clinically relevant information on patient demographics, attendance location and characteristics, clinical signs, treatment details, and outcomes. Validation rules, including mandatory fields that block progression until complete, and time-stamps to ensure details cannot be completed before attendance arrival, are built into the *e*PCR system, resulting in highly detailed and complete *e*PCRs. NASS is based entirely on coding these clinical records without placing any additional workload on paramedics. Prior to 2006, the Victorian ambulance service used paper-based records.

#### Step 2 –Filtering

Three of the six ambulance services (New South Wales, Queensland, and Victoria) undertake primary filtering prior to providing the *e*PCR to NASS. The filter (S1 File) is based on an automated keyword search to extract cases that potentially involve AOD consumption, using over-inclusive parameters to maximise probable case capture. VACIS^®^ case capture, data matching and filtering have been previously described [24], and the filtering used in this project follows a similar process. Filtering is dynamic with manual reviewing in response to discrepancies in the project extract and comparison with other data systems, however cases may be missed, resulting in an underestimation of AOD-related cases. The remaining three ambulance services (Australian Capital Territory, Northern Territory and Tasmania) provide unfiltered priority 1 and 2 attendances.

#### Step 3 –Initial processing at Turning Point

Jurisdictional ambulance services extract case records from their data management systems and securely transfer these *e*PCRs to Turning Point. On receipt, data are checked for completeness and compared to the Turning Point database. Graphs of attendance numbers are created as a visual check for anomalous trends. Discrepancies are flagged for systematic investigation by the database manager. Once *e*PCRs pass all checks, they are appended to the data warehouse and prepared for manual coding.

#### Step 4 –Coding: Case ascertainment and case classification

Coding occurs in a purpose-built system by a team of research assistants (RAs) who manually scrutinise each *e*PCR to determine whether the case meets core inclusion criteria (case ascertainment) as well as to identify the substances involved in the attendance (case classification). The time to code a single ambulance attendance ranges from 1.28 to 4 minutes, and each RA codes approximately 180 attendances per day. Workflow is managed with the use of a data warehouse, with allocation of records based on reporting priorities.

Inclusion criteria are met if recent, inappropriate AOD use contributed to the ambulance attendance, using the following criterion: ‘Is it reasonable to attribute the immediate or recent (the past 24-hours) over or inappropriate AOD use as a contributing reason for the ambulance attendance?’ This information is ascertained from paramedic clinical assessment, patient self-report, information from third parties and evidence at the scene, as recorded in the clinical notes. Importantly, while AOD consumption must be a contributor to the ambulance attendance, it may not be the primary reason for the ambulance attendance.

Case classification occurs at the level of individual substances, including alcohol, 13 illicit drugs (individual substances presented in Table 2) and 82 pharmaceutical medications (individual medications presented in S1 Table), with each listed substance coded and routinely reported. For pharmaceutical medications (including over-the-counter), inappropriate use is defined as consumption contradictory to prescriber or manufacturer instructions. Specifically, consuming these substances in excess of the prescribed dose, drugs prescribed for another person, and/or consumption in combination with contraindicated substances is deemed inappropriate use. Adverse events following appropriate medication use are excluded. ‘Other pharmaceutical medication’ classification includes all substances prepared in pharmaceutical settings not further specified by codes, including over-the-counter medications not elsewhere specified, vitamins, and herbal supplements. For illicit substances, any consumption is classified as AOD-related. Substances not intended for human consumption and not captured elsewhere are classified as ‘other substances’.

**Table 2:** National Ambulance Surveillance System output variables, including scene patient and clincal details, alcohol, individual illicit drugs and pharmaceutical medication drug classes*.

Case details	Patient details	Scene details	Physical condition	Illicit drugs	Pharmaceutical medications	Other substances	Intent of AOD poisoning
Case number	Gender	Public / private	Patient outcome	Methamphetamine	Opioid analgesics	Alcohol involved	Unintentional
Case date	Age	Indoor / outdoor	Pulse rate	Crystal methamphetamine	Other analgesics	Alcohol intoxication	Intentional
Case time	Residential postcode	Event postcode	Blood pressure	Cannabis	Benzodiazepines	Inhalant	Undetermined intent
Transport to hospital	Homelessness	Event coordinates	Respiratory rate	Synthetic cannabinoids	Anti-depressants	Other substance	
Reason for not transporting	Unemployment	Police co-attendance	Skin temperature	Emerging psychoactive substances	Anti-psychotics		
	Previous incarceration	Others on scene	Skin moisture	Cocaine	Anti-convulsants		
	Culturally and linguistically diverse	Minors on scene	Skin colour	3,4-methylenedioxy-methamphetamine (MDMA)	Opioid pharmacotherapy treatments		
	Refugee background		GCS eye response	Gamma hydroxybutyrate (GHB)	Pharmaceutical stimulants		
			GCS verbal response	Heroin	Peer administered naloxone		
			GCS motor response	Ketamine	Other medication		
			Naloxone administration	Lysergic acid diethylamide (LSD)			
			Naloxone dose	Mushrooms			
			Naloxone response	Other illicit drugs			

*Individual pharmaceutical drugs presented in S1 Table

Most individual substances are simply identified as ‘related’ to the ambulance attendance. The exceptions are alcohol, heroin, and pharmaceutical opioids. Attendances with any alcohol consumption, ranging from small (i.e., <1 standard drink) to large quantities are classified as ‘alcohol involved’, which is particularly useful when examining effects of possible interactions with other drugs. As blood alcohol levels are not completed by paramedics, an ‘alcohol intoxication’ proxy measure was devised. This distinction is based upon paramedic clinical assessment of intoxication, supported by the reported alcohol quantity consumed. Like other health professionals, paramedics undergo significant training to recognise the signs and symptoms of overdose, particularly for more common substances, in order to respond and provide treatment to the patient. This clinical knowledge is supplemented by their ability to draw on additional evidence available on scene that is not available in hospital or other clinical settings. For example, alcohol bottles, injecting equipment, medication bottles/vials/packets, friends, family and associates who can provide additional information, as well as signs and symptoms ensure that the clinical documentation of the case contains reliable information on substances involved in the attendance. Cases involving naloxone administration by paramedics with a positive response are classified as naloxone-responsive opioid-related cases. These are further defined as ‘heroin-‘, ‘specific pharmaceutical preparation-‘, or ‘unknown opioid-‘related cases.

To indicate collective impact of AOD consumption on an ambulance attendance, a supplementary classification of AOD use was defined and coded: unintentional AOD poisoning (overdose threshold met). Case inclusion criteria varies depending on the type of drug consumed, using proxy measures to identify cases with potential medical harms: (a) alcohol and/or illicit substances: potentially life threatening, identified by a clinical case involving a Glasgow Coma Scale (GCS) score of less than nine [25], low respiratory rate and/or paramedic concern for securing an airway; (b) pharmaceutical medications: concordant clinical picture of alcohol or illicit drug AOD poisoning, or the consumption of 10 or more times the typically prescribed dose for the specific preparation concerned. Case inclusion criteria for pharmaceutical drugs varied from that of alcohol and illicit substances due to the complexity of considering total drug effect for individual pharmaceutical medications, during the manual coding process. Case inclusion criteria also apply to coding categories used in the self-harm component of NASS: intentional AOD poisoning (AOD consumption with suicidal intent) and undetermined intent AOD poisoning (determination of intentional or unintentional AOD poisoning cannot be made); however, further explanation of these coding categories is beyond the scope of the present paper.

#### Step 5 –Data review and export

After a set of *e*PCRs are manually coded, the dataset is reviewed by project staff and extracted for data cleaning. The cleaning process is systematic, and each step must be completed before progression. A selection of summary variables are checked for missing data, and multiple *e*PCRs for the same patient are aggregated and duplicates removed. Data are then converted to a format suitable for analysis, exported, merged with the master dataset, and backed up. During this process, summary information from the data warehouse and master dataset are scrutinised to verify accuracy of the export process. Any unusual or unexpected results identified during analysis are re-reviewed to ensure data accuracy.

### Coder training and validation

RAs undertake extensive, iterative training to ensure appropriate (a) case ascertainment and (b) case classification. RAs use coding rules and guidelines that detail the inclusion criteria, classification process, define case classification, and provide examples of common and uncommon attendances. New RAs are trained by senior researchers, and then paired with multiple experienced RAs on a rotating basis, until they and their senior coding partner are confident of coding quality. Senior researchers review records coded by new RAs to ensure inter- and intra-coder reliability. Following database changes (e.g., introduction of new variables), RAs are re-trained via workshops and “dummy case exercises”. Workshops are also used to identify coding difficulties, develop and test operational solutions and disseminate coding clarifications on an ongoing basis. If RAs are not confident in assigning codes for a specific attendance, the attendance is escalated to a senior researcher for review (this occurs in <1% of attendances), rules and guidelines clarified accordingly and disseminated to the coding workforce.

Coding audits are conducted on a routine bases to ensure inter-coder reliability. These provide a learning tool for RAs, and parameters may change over time based on the relative experience of the current coding team and introduction of new coding modules. Results of a recent coding audit are described. A maximum of 90 previously coded attendances per RA were extracted, from the Victoria Quarter 1 2017 dataset. The 90 attendances met 26 criteria for case classification. Each RA then re-coded a random selection of records where they were not the original coder. At that time, there were 23 individual RAs, and the records they re-coded came from an average of 9±3 (mean±standard deviation) other RAs. A total of 1,718 attendances were re-coded, which meant re-coding of 221,622 AOD variables, of which only 470 differences were identified. Differences were systematically identified, and reviewed by a senior researcher for personalised feedback to each RA and then followed up with systematic team training.

### Analysis

Project success remains contingent on active partnerships with jurisdictional ambulance services. Senior staff adopt a collaborative and inclusive approach, ensuring project buy-in and commitment by implementing: (a) transparent data use processes communicated by routine reporting; (b) formal agreements to ensure data provision, analysis and reporting meet ambulance service requirements; (c) consistent opportunities for contribution, feedback and review of outputs; and (d) formal acknowledgement of partnerships in dissemination.

Time lags for data transfer vary across jurisdictional ambulance services, with most data available for coding between one-to-three months from the end of the data collection period. Data processing and coding at Turning Point ensures data consistency, quality, and timeliness. Generally, manual coding can be completed within one to two months of data acquisition, depending on the number of attendances and project reporting requirements.

### Output variables

NASS captures more than 140 variables, including patient and scene details, physical condition of patient and substances consumed. Ethnicity is collected within the raw ambulance service data for some jurisdictions, however it is not collected reliably on a national basis and we do not report it. Not all ambulance services collect details on ethnicity as the time they are with the patient is limited and the details of ethnicity are often not clinically relevant to stabilising and treating the patient. Extensive sociodemographic variables are collected and coded, such as homelessness, unemployment, previous incarceration, and culturally and linguistically diverse and refugee backgrounds (Table 2).

Ambulance attendances are coded to the level of the individual substance, which is not routinely captured by other population-level data (for example, hospital and emergency department data). Output variables are summarised in Table 2. The column of pharmaceutical medications can be expanded into 82 individual commonly used medications within the broader drug classes presented (S1 Table). The surveillance system is flexible in that specific drugs can be added as they become of interest. Of note, some variables are not available from all ambulance systems (e.g. event coordinates by GPS only available from New South Wales and Victoria) due to jurisdictional system capacity and project agreements.

### Relevant cases identified at each process step

Relevant cases identified at each step of the NASS process, using 12 months of Victorian data (2016–17 financial year), are presented in Table 3. The total rate of case ascertainment across the five steps of the process in this example year was 10.0%.

**Table 3:** Identification of relevant cases through the NASS process, Victoria, 2016–2017 financial year*.

Process step	Number of ambulance attendances	Inclusion rate from preceding step
1 –Ambulance data collection	497,814	N/A
2 –Provided to Turning Point after filtering	128,641	25.8%
3 –Proceeded to coding after initial data processing	128,641	100.0%
4 –Case ascertainment (deemed to be AOD-related)	50,685	39.4%
5 –Available for analysis after data export	49,647	98.0%

*Australian financial year, from 1 July 2016 to 30 June 2017

Cases that do not meet inclusion criteria for AOD consumption at Step 4 are not discarded, but may be classified for inclusion in other components of NASS, including those focussing on mental health, self-harm or violence.

### Demonstration of data utility

**Fig 2:**
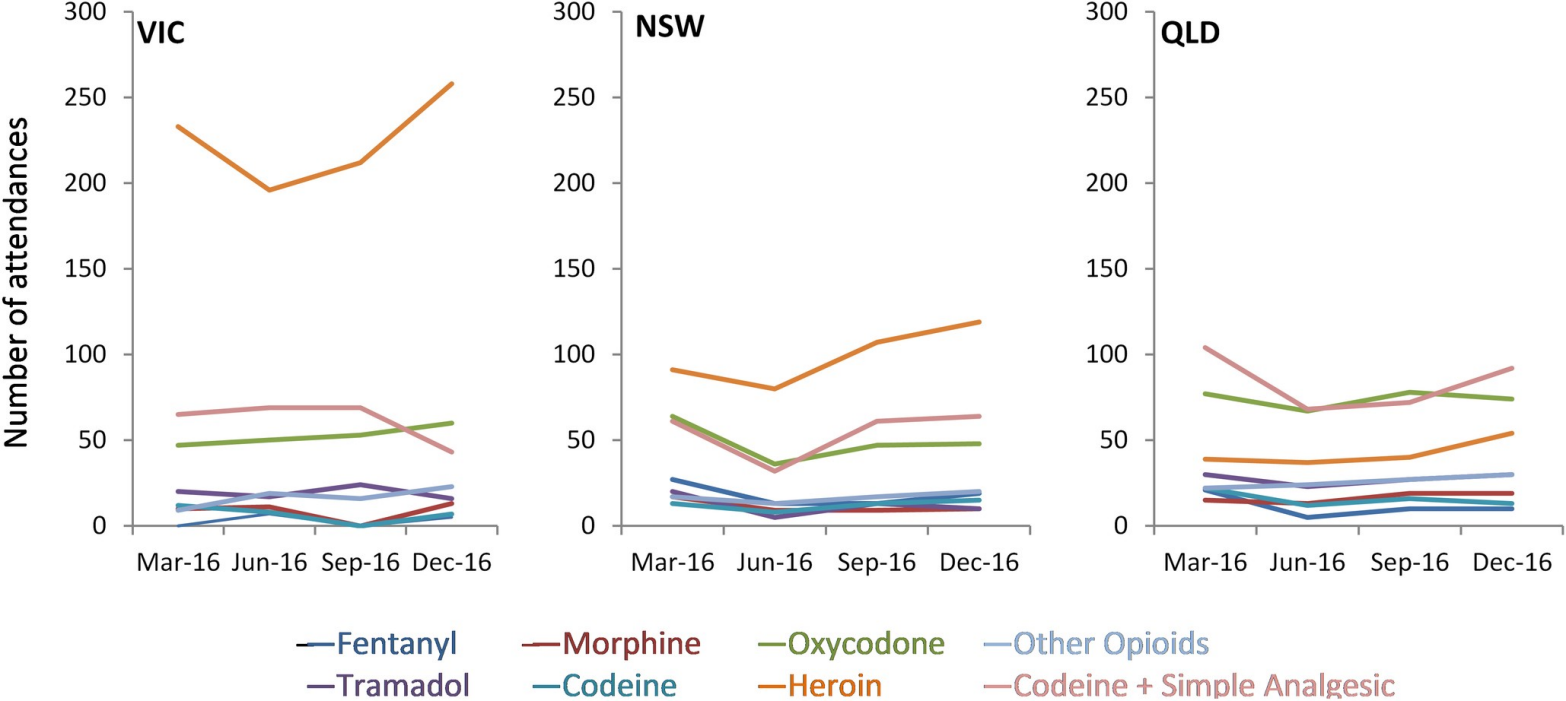
Opioid-related ambulance attendances by jurisdiction, March, June, September and December 2016. Australian Capital Territory, Northern Territory and Tasmania not presented due to small numbers.

Fig 2 highlights capacity of NASS to analyse trends for individual substances and medications not captured in other population-level AOD data, and presents opioid-related ambulance attendances from the three largest jurisdictions (New South Wales, Queensland and Victoria). These features have important policy implications. For example, data have been used as supporting evidence in the selection of preparations to include in *SafeScript*, the real-time prescription monitoring program recently implemented in Victoria [26], as well as the evaluation of lock-out laws in Sydney’s entertainment districts [27].

Additionally, data have been used to elucidate gender and age affects in the context of attendances for AOD harms. Examples include examination of inhalant-related harms in young females, a group that is traditionally underrepresented in AOD research, with the majority of research to date relating only to young males [28]. Acute harms relating to AOD use and co-occurring self-harm and mental health symptomology in children under 12 years have also been described and compared with an older cohort, addressing a knowledge gap as people under 12 are excluded from routine survey research [29].

## Results and discussion

We describe a novel method of monitoring, analysing and reporting acute AOD-related harms at a population level, using coded ambulance clinical records. Importantly, this project provides consistent, detailed, and timely data with spatial and temporal analysis capacity for AOD-related harms not captured by other systems. This project has been successfully established, with partnerships built across Australian ambulance services, to provide a national surveillance system of acute AOD-related harms with comprehensive coverage that includes diverse and hard-to-reach groups, such as drug-using populations, youth who are not captured by survey data or those experiencing acute issues that only present at a time of crises.

**Fig 3:**
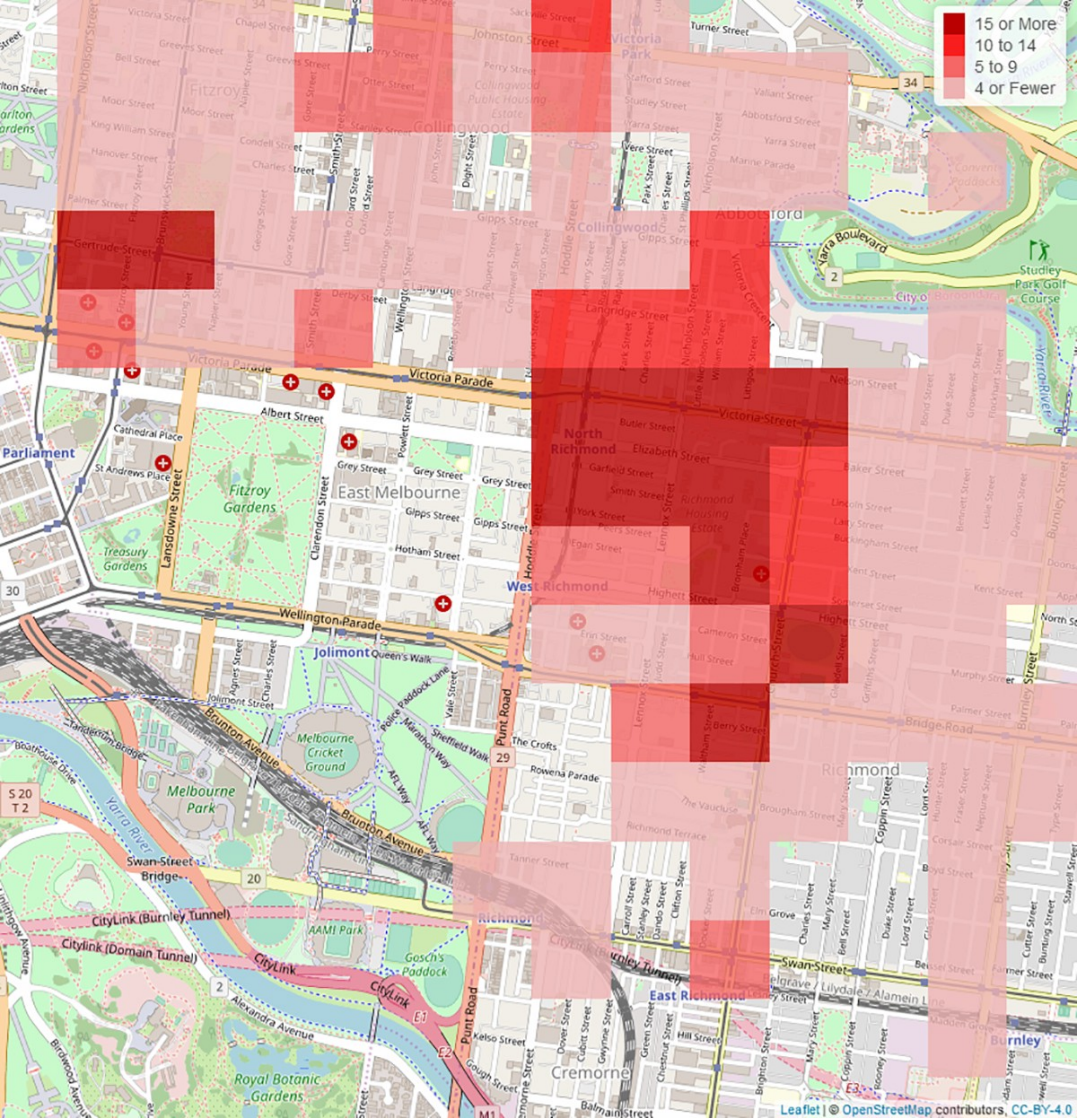
The number of opioid-related ambulance attendances in one local government area in metropolitan Melbourne. Ambulance attendances are shown within 250 metre squares, based on GPS data. The map source information is provided by OpenStreetMaps contributors.

There are multiple benefits of undertaking monitoring in this way. NASS uses coded patient clinical notes from the *e*PCR, therefore there are no recruitment or self-selection biases, and there is no added workload for paramedics. A centralised coding system at Turning Point is efficient, timely, reliable and accurate and does not require duplication across jurisdictions. Reporting is highly specific with regards to substance type, and therefore policy, intervention and practice changes can be informed by examining emerging trends and employing a public health approach. NASS provides an internationally unique resource that sits alongside population measures of AOD use and burden of disease modelling, to provide an understanding of the impacts of AOD consumption. Data from NASS have informed public policy interventions and clinical practice related to AOD trends and harm [28–32]. Additionally, Victorian data are freely accessible and used by the public, policy makers, clinicians, and researchers via an online interactive website (www.AODstats.org.au). Spatial and temporal data can be used to inform service provision requirements, with highly granular data available (Fig 3). These data have clinical utility, and have been used to identify emerging trends related to the misuse of prescription medications (e.g., quetiapine [33] and pregabalin [34]), illicit drugs (e.g., GHB) [35] as well as mental health harms (e.g., methamphetamine in psychosis-related ambulance attendances [36]). Data can also be used to highlight population sub-groups at particular risk of harm, who may be hard-to-reach (e.g., substance use in young adolescents) [29]. Data has also been used to compare patterns of harms in substances and patterns of substances in specific harms [37].

In terms of limitations, the data do not capture attendances related to chronic harms; data only relate to acute AOD harms where an ambulance is called and attends. Like other coded health datasets, NASS is reliant on clinical information collected for operational rather than research purposes, with the potential for incomplete, inaccurate, or inconsistent recording of variables impacting data quality. Primary filtering of data may exclude cases of interest; however the filters are set to be intentionally broad to improve capture of relevant cases, and the potential for missing cases means that coded NASS data are conservative and under-estimate the number of AOD-related cases. Extensive *e*PCR scrutiny by trained staff coupled with collaborative communication between project staff and ambulance services is undertaken to minimise data inconsistencies or irregularities. Additionally, although ambulance services are considered universal in Australia, there are financial and geographic barriers to access in very remote areas of the country. NASS has been possible in Australia due to the nature of ambulance services providing a universal service at a state and territory level, covering a large proportion of the population using the same or similar *e*PCR systems. Other countries may face challenges introducing such a system due to large numbers of small ambulance services, lack of common *e*PCR systems, dependence on paper-based systems, or financial barriers to access.

Efforts to include the remaining two Australian jurisdictions are ongoing. A trial to code South Australia’s data found ongoing inclusion was unworkable due to additional resources associated with their paper-based patient clinical record system. Negotiations are expected to recommence when an *e*PCR system becomes operational in South Australia. Data provision from Western Australia commenced in late 2018, and comparable data provision is anticipated in 2020.

There are opportunities to improve NASS’s utility. Like the Australian hospital admission dataset, the NASS data presented are coded on the basis of episodes of care rather than individual patients. Although tracking individuals had not been possible, our data has accurately represented total service burden. A process is nearing completion for Victorian and NSW data to use a SLK to enable analysis by individuals rather than attendances. This SLK also enables linkage across health datasets, to track an individual’s trajectory through the health system to inform: (a) their treatment needs; (b) service provision; and (c) longer term outcomes, including death. This dataset will be improved by continuous coverage across jurisdictions other than Victoria, and inclusion of the remaining two Australian states. A target of future research is to develop rapid reporting methods, artificial intelligence and natural language processing to improve coding efficiencies so that emerging issues and patterns can be identified and reported faster. Coding for commonly co-occurring issues [2,38], such as mental health, violence, and self-harm, are conducted in complimentary modules of the project, and add significantly to the value of the data by capturing the nature and context of such presentations. In summary, we have demonstrated the utility of an internationally unique approach to quantify and monitor acute AOD-related harms in the community, providing a methodology that other countries and jurisdictions could adopt.

## Conclusions

Excessive consumption of alcohol and illicit drugs, and extra-medical use of pharmaceutical medications, are major avoidable risk factors for disease, illness, injury and death. To understand the impact of AOD consumption and develop public health responses, it is important that related harms are measured. Coded ambulance attendance data offers a timely source of information on harms that may be missed in other datasets. This paper describes NASS, an internationally unique surveillance system that captures acute harms related to individual drugs using coded ambulance clinical records, reporting temporal and spatial trends within months of their occurrence. NASS has been used to inform and evaluate policy approaches and potential points of intervention at the local and national level, as well as guide workforce development needs and clinical practice.

## Supporting information

S1 File Filtering of electronic patient care records prior to coding in the National Ambulance Surveillance System. (PDF)

S1 Table Individual pharmaceutical medications coded in the National Ambulance Surveillance System. (PDF)
