# Supplementary material for: The National Ambulance Surveillance System: A novel method for monitoring acute alcohol, illicit and pharmaceutical drug related-harms using coded Australian ambulance clinical records
Source: PLoS One. 2020 Jan 31;15(1):e0228316. doi: 10.1371/journal.pone.0228316 (PMC6994147; doi:10.1371/journal.pone.0228316)
Supplement: S1 File — (PDF) [file pone.0228316.s001.pdf]

S1 File Filtering electronic patient care records prior to coding in the National Ambulance Surveillance System

The case records received by Turning Point from the ambulance service providers are a filtered selection from the total number of cases attended-to by the respective service provider. Each service provider performs a different degree of filtering[1], from basic elimination of scheduled transfers and aborted call outs, to complex filtering based on case type.

The original filtering algorithm ("the Filter") was developed by Ambulance Victoria based on the VACIS® database during the initial establishment of the Ambo Project (formerly known as Turning Point Overdose Study (TPOS)), with input from Turning Point regarding cases of interest. As the Filter was developed for a large scale proprietary system, the precise details are known only to Ambulance Victoria. However, as other jurisdictional ambulance services, notably Queensland and New South Wales, also used VACIS®[2], the Filter was passed on to assist these states' ambulance services to develop their own filtering algorithms. As VACIS® has been upgraded, and as the scope of the Ambo Project and National Ambulance Surveillance System expanded, the Filter has been updated over time to reflect these changes.

It is important to note that the extract files and tables received from ambulance services, even those using the same version of VACIS®, differs substantially between ambulance services. Further, the structure of these tables are not identical to VACIS®. Turning Point does not have access to the table structures and fields in VACIS® (or any other systems'), and therefore the following details of the Filter are of generic guidance only based on the tables received by Turning Point.

In Victoria, cases for National Ambulance Surveillance System are selected based on the following criteria (at least one match):

- Case nature includes: "Inhalation", "Overdose", "Alcohol", "Drug", "Mental Health", "Emotional Problem", "Psychiatric Problem", or "Social Situation";

[1] Ambulance Victoria, NSW Ambulance and Queensland Ambulance Service perform extensive filtering of cases prior to sending them through to Turning Point. Australian Capital Territory Ambulance Service filters out scheduled, non-acute cases. Ambulance Tasmania formerly filtered out scheduled non-essential cases, however this has been conducted by Turning Point since January 2017. St John Ambulance Australia (NT) does not apply a filter to cases.
[2] Ambulance Victoria, NSW Ambulance, Australian Capital Territory Ambulance Service and Ambulance Tasmania use their own instances of VACIS®. Queensland Ambulance Service used their own instance of VACIS® until end of 2017 and have transitioned to a Queensland specific system. St John Ambulance Australia (NT) uses the Siren® ePCR system.

- Complaint includes: “Alcohol”, “Drug Withdrawal”, “Anxiety”, “Behavioural”, “Hallucinations”, “Depression”, “Insomnia”, “Panic”, “Self Harm”, “Spinning Out”, “Suicidal”, “Suicide Attempt”, or “Emotional Distress”;
- Survey includes: “Alcohol”, “Drug Paraphernalia”, or “Injection Marks”;
- Assessment includes: “Alcohol”, “Drug”, “Overdose”, “Anxiety”, “Depression”, “Eating Disorder”, “Emotional Distress”, “Panic Attack”, “Psychiatric Episode”, “Sleep Disorder” or “Social Problem”;
- Naloxone Hydrochloride given or drawn;
- A mental health assessment shows hallucinations, delusional thoughts, depressive thoughts, suicidal thoughts or bizarre behaviour;
- Event type is: overdose, poisoning, psychiatric, or mental health;
- Billing type is mental health or psychiatric;
- Psychiatric services are on the scene;
- A mental health procedure was documented;
- The case description includes: “psych”, “mental”, “suicide*”, “self harm”, transportation under the Mental Health Act, mention of a Community Treatment Order, or mention of Crisis Assessment and Treatment.

Each state applies their filters (if any) using their own method to suit their unique system. Turning Point does not perform additional filtering prior to coding cases, except where noted or required for specific projects (e.g. by gender or scene location).
