# Supplementary material for: The National Ambulance Surveillance System: A novel method for monitoring acute alcohol, illicit and pharmaceutical drug related-harms using coded Australian ambulance clinical records
Source: PLoS One. 2020 Jan 31;15(1):e0228316. doi: 10.1371/journal.pone.0228316 (PMC6994147; doi:10.1371/journal.pone.0228316)
Supplement: S1 Table — (PDF) [file pone.0228316.s002.pdf]

S2 Table Individual pharmaceutical medications coded in the National Ambulance Surveillance System

**Opioid analgesics**	**Other analgesics**	**Benzodiazepines**	**Anti-depressants**	**Anti-psychotics**	**Anti-convulsants**	**Opioid pharmacotherapy treatments**	**Pharmaceutical stimulants**
Dextropropoxyph ene	Aspirin	Alprazolam	Amitriptyline	Amisulpride	Carbamazepine	Buprenorphine	Atomoxetine
Fentanyl	Aspirin & Codeine	Bromazepam	Citalopram	Aripiprazole	Gabapentin	Buprenorphine & Naloxone	Dexamphetamine
Hydropmorphone	Codeine	Clonazepam	Clomipramine	Chlorpromazine	Lamotrigine	Methadone	Methylphenidate
Morphine	Ibuprofen	Diazepam	Desvenlafaxine	Clozapine	Pregabalin	Naltrexone	Other
Oxycodone	Ibuprofen & Codeine	Flunitrazepam	Dothiepin	Fluphenazine	Sodium valproate		
Oxycodone & Naloxone	Paracetamol	Lorazepam	Doxepin	Haloperidol	Tiagabine		
Pethidine	Paracetamol & Codeine	Midazolam	Duloxetine	Lithium	Topiramate		
Tramadol	Other	Nitrazepam	Escitalopram	Olanzapine	Other		
Other		Oxazepam	Fluoxetine	Paliperidone			
		Temazepam	Fluvoxamine	Pericyazine			
		Zolpidem	Imipramine	Quetiapine			
		Zopiclone	Mianserin	Risperidone			
		Other	Mirtazapine	Trifluoperazine			
			Moclobemide	Ziprasidone			
			Nortriptyline	Zuclopenthixol			
			Paroxetine	Other			
			Reboxetine				
			Sertraline				
			Venlafaxine				
			Other				
